# Ether Lipid Deficiency in Mice Produces a Complex Behavioral Phenotype Mimicking Aspects of Human Psychiatric Disorders

**DOI:** 10.3390/ijms20163929

**Published:** 2019-08-13

**Authors:** Fabian Dorninger, Anna Gundacker, Gerhard Zeitler, Daniela D. Pollak, Johannes Berger

**Affiliations:** 1 Department of Pathobiology of the Nervous System, Center for Brain Research, Medical University of Vienna, Spitalgasse 4, 1090 Vienna, Austria; 2 Department of Neurophysiology and Neuropharmacology, Center for Physiology and Pharmacology, Medical University of Vienna, Schwarzspanierstraße 17, 1090 Vienna, Austria

**Keywords:** plasmalogen, ether phospholipid, autism, behavior, peroxisome, social interaction, memory, neurotransmission, synapse, Alzheimer’s disease

## Abstract

Ether lipids form a specialized subgroup of phospholipids that requires peroxisomes to be synthesized. We have previously detected that deficiency in these lipids leads to a severe disturbance of neurotransmitter homeostasis and release as well as behavioral abnormalities, such as hyperactivity, in a mouse model. Here, we focused on a more detailed examination of the behavioral phenotype of ether lipid-deficient mice (*Gnpat* KO) and describe a set of features related to human psychiatric disorders. *Gnpat* KO mice show strongly impaired social interaction as well as nestlet shredding and marble burying, indicating disturbed execution of inborn behavioral patterns. Also, compromised contextual and cued fear conditioning in these animals suggests a considerable memory deficit, thus potentially forming a connection to the previously determined ether lipid deficit in human patients with Alzheimer’s disease. Nesting behavior and the preference for social novelty proved normal in ether lipid-deficient mice. In addition, we detected task-specific alterations in paradigms assessing depression- and anxiety-related behavior. The reported behavioral changes may be used as easy readout for the success of novel treatment strategies against ether lipid deficiency in ameliorating nervous system-associated symptoms. Furthermore, our findings underline that ether lipids are paramount for brain function and demonstrate their relevance for cognitive, social, and emotional behavior. We hereby substantially extend previous observations suggesting a link between deficiency in ether lipids and human mental illnesses, particularly autism and attention-deficit hyperactivity disorder.

## 1. Introduction

Aberrant behavior and/or personality changes are symptoms of severe psychiatric diseases including neurodevelopmental disorders, such as autism spectrum disorders (ASD) or attention-deficit hyperactivity disorder (ADHD). Although the molecular details involved in the pathophysiology of these diseases are the subject of ongoing research, it is undisputed that alterations in synaptic transmission (also termed neurotransmission) are critically involved [1,2]. Neurotransmission is a highly complex procedure involving the coordinated interplay of a multitude of molecular players. Mutations in a large variety of genes encoding synaptic components, for example, proteins involved in the synaptic vesicle cycle, metabolic enzymes, postsynaptic receptors, scaffolding proteins, and many others, have been associated with psychiatric and neurodevelopmental disorders. However, the genetics of these illnesses is usually non-Mendelian and intricate and mostly defines vulnerability, disease development, and disease course within a complex web of gene/environment interactions. Apart from the proteins directly involved in synaptic transmission, it has more recently come to light that also the lipid environment of the participating compartments is a major modulatory factor for neurotransmission [3,4,5] and changes in lipid composition have been frequently linked to neurologic and psychiatric diseases [6,7,8,9,10]. Our latest work has elucidated ether lipids, a subclass of phospholipids, as important components impacting neurotransmitter homeostasis and release [11]. These compounds are structurally characterized by an ether bond at the *sn*-1 position of the glycerol backbone and can be classified into various subgroups, among which the group of so-called plasmalogens is by far the most abundant [12]. Ether lipids are indispensable for humans, as demonstrated by the severe consequences of an inborn deficiency in ether lipid biosynthesis evoking the fatal disorder rhizomelic chondrodysplasia punctata (RCDP). Even though the molecular details of ether lipid biology have not been fully clarified, it is now established that these lipids perform essential roles in shaping membrane integrity and properties, thus potentially influencing countless biological processes, as well as crucial messenger functions [13]. Also, antioxidative capacities have been ascribed to plasmalogens [14,15]. Remarkably, some patients with RCDP, particularly those with less severe forms of the disease and showing extended survival, display behavioral anomalies, i.e., features of neurodevelopmental disorders like ADHD or ASD [16]. Furthermore, a genetic association of *PEX7*, one of the genes involved in the etiology of RCDP, with ASD has been reported [17]. Our previous data involving hypothesis-driven testing of specific behavioral features in a mouse model of complete ether lipid deficiency, the *glycerone phosphate O-acyltransferase* knockout (*Gnpat* KO) mouse, have indicated a behavioral phenotype reproducing certain aspects of human neurodevelopmental disorders. Specifically, although dealing with widespread physical impairments including ocular deficits, male infertility [18], central and peripheral hypo- and dysmyelination [19,20], and a dysfunctional neuromuscular junction [21], ether lipid-deficient mice are hyperactive and exhibit impaired social behavior [11]. 

In the current study, we built upon these data and set out to examine in greater detail the behavioral phenotype of mice with genetically determined ether lipid deficiency, with a focus on behaviors relevant for neurodevelopmental and psychiatric disorders. Together with our previous studies [11,21], these data provide a comprehensive overview of the neurobehavioral phenotype of ether lipid-deficient mice, thus adding to our understanding of the molecular players involved in neurologic diseases and offering a potential simple readout for therapeutic approaches tackling ether lipid deficiency. 

## 2. Results

### 2.1. Ether Lipid-Deficient Mice Show Markedly Impaired Social Interaction but no Apparent Changes in the Preference to Social Novelty

**Figure 1:**
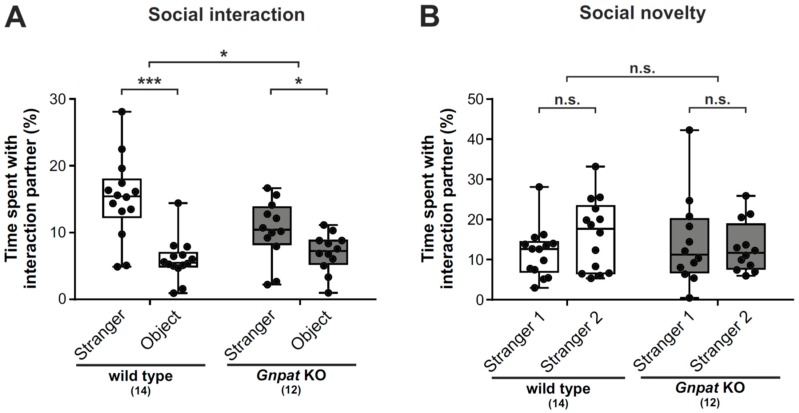
*Gnpat* knockout (KO) mice show deficient social interaction but no apparent changes in the response to social novelty: A three-chamber approach was used to assess sociability (**A**) and the response to social novelty (**B**). In the social interaction task, the time spent interacting with an unfamiliar mouse (“stranger”) or a neutral, novel object (“object”) was quantified automatically. For the evaluation of social novelty, mice were exposed to a previously encountered mouse (“stranger 1”) in one chamber and a completely unfamiliar mouse (“stranger 2”) in the opposite chamber. The time spent with each of the strangers was quantified automatically. Data are shown as box plots together with individual data points, and whiskers indicate minimal and maximal values. The number of tested animals is given in brackets. Statistical analysis was performed using paired, two-tailed Student’s *t*-tests (for the comparison of the preferences within each genotype) and repeated measures two-way ANOVA (for comparison of the genotypes). The findings displayed in Figure 1A have been published previously in a smaller cohort of wildtype (WT) and *Gnpat* KO mice [11]. *** *p* < 0.001, * *p* < 0.05; *n.s.*, not significant.

We previously reported that ether lipid-deficient mice exhibit significant deficits in social interaction [11]. Here, we extend these findings by (i) confirming the previous data in a larger cohort of animals and (ii) by including an additional paradigm addressing the response to social novelty. We investigated social interaction using a three-chamber approach, where sociability is characterized by the test mouse spending significantly more time in the chamber with a stranger mouse than in the chamber with a neutral object (black Lego^®^ block) [22]. Consistent with this definition, wildtype (WT) mice exhibited a considerable preference for the stranger mouse in our setting (Figure 1A). However, sociability was clearly reduced in *Gnpat* KO mice, which spent only slightly more time interacting with the stranger than with the inanimate object (Figure 1A).

To study the response to social novelty, the inanimate object was subsequently replaced by another mouse (“stranger 2”). Most mouse strains have been described to show a preference for social novelty, i.e., stranger 2 over stranger 1 [23]. While there was only a slight, statistically nonsignificant trend towards longer interaction times with stranger 2 for WT mice (Figure 1B), no such trend was observed for ether lipid-deficient mice.

Testing for sociability and social novelty heavily relies on olfactory cues, but correct functioning of the olfactory system has not been examined previously in ether lipid-deficient mice. In order to control for this factor, we compared performance in the buried food test between WT and *Gnpat* KO mice. Even though the median latency to find and consume a buried food pellet was longer in ether lipid-deficient mice, we did not find any statistically significant difference between the two genotypes indicating roughly normal olfaction in ether lipid-deficient animals (Figure S1).

### 2.2. Ether Lipid-Deficient Mice Exhibit Substantial Deficits in Marble Burying Behavior

**Figure 2:**
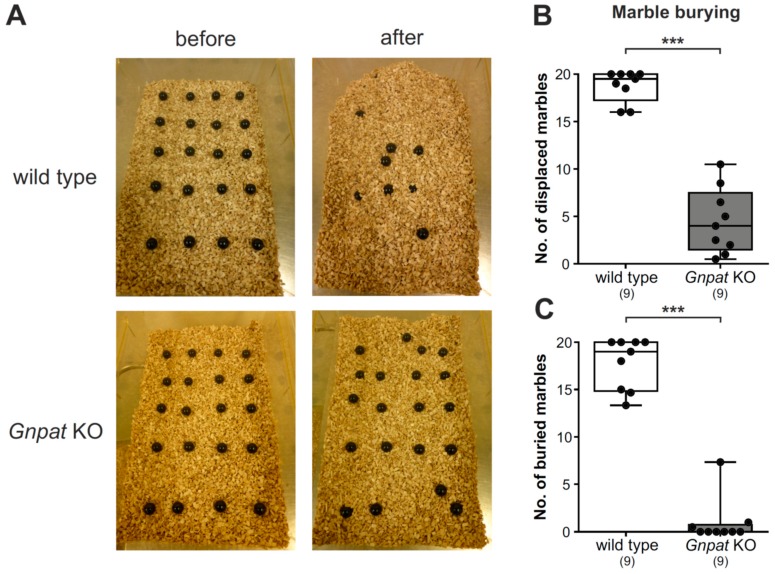
Striking alterations in the marble burying assay in ether lipid-deficient mice: Marble burying behavior was examined in WT and *Gnpat* KO mice, and representative pictures before and after 30 min trial time are shown (**A**). The number of marbles displaced (**B**) and buried (**C**) were quantified. Data are shown as box plots together with individual data points, and whiskers indicate minimal and maximal values. The number of tested animals is given in brackets. Statistical analysis was performed using the Mann–Whitney U test. *** *p* < 0.001.

Marble burying as an indicator of repetitive behavior is routinely used for the characterization of autism-related behavior [24,25]. As expected, based on previous reports [26], WT mice displaced and buried a considerable number of marbles by repeated digging during the trial time (Figure 2A, Video S1). However, *Gnpat* KO animals did not at all engage in digging behavior, leaving most of the marbles in their place. Consequently, the quantification of the number of marbles displaced (Figure 2B) or buried (Figure 2C) yielded large and statistically highly significant differences between the genotypes. However, ether lipid-deficient animals clearly registered and interacted with the marbles (e.g., by sniffing or trying to bite them; Video S2), which excludes the hypothesis that *Gnpat* KO mice simply did not note the presence of the marbles.

### 2.3. Normal Nesting Behavior but Reduced Nestlet Shredding in Gnpat KO Mice

The assessment of nesting behavior is a routine test in mice with genetic modifications, and impaired nesting has been previously reported in mouse models characterized by autistic traits like impaired social interaction [27,28,29]. Thus, we provided WT and *Gnpat* KO mice with commercially available nestlets and monitored nest building at two time points (20 and 48 h). Mice of both genotypes readily utilized the nestlets (Figure S2A), and there was no statistically significant difference in median scores for nest quality at both examined time points (Figure S2B,C).

**Figure 3:**
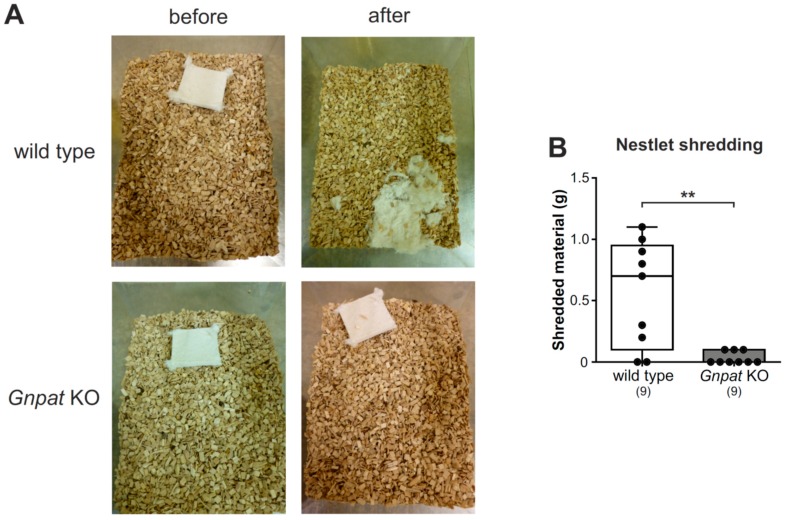
Reduced nestlet shredding in *Gnpat* KO mice: Nestlet shredding was evaluated in WT and *Gnpat* KO mice, and representative pictures are shown (**A**). The weight reduction of the nestlet after the trial was quantified, and the results are shown as box plots together with individual data points, and whiskers indicate minimal and maximal values (**B**). The number of tested animals is given in brackets. Statistical analysis was performed using the Mann–Whitney U test. ** *p* < 0.01.

In another setting, we examined nestlet shredding behavior by providing the mice with nestlets for an observation period of 30 min and by recording the weight of the shredded nestlet material. Here, in spite of marked variability, most of the WT mice processed parts of the nestlet (Figure 3A). For *Gnpat* KO mice, the amount of shredded material was statistically significantly reduced (Figure 3B), and in several cases, the mice did not engage in processing the nestlet at all.

### 2.4. Gnpat KO Mice Show Alleviated Behavioral Patterns of Anxiety in the Elevated Plus Maze and the Light/Dark Box Test but Not the Novelty-Suppressed Feeding Test

**Figure 4:**
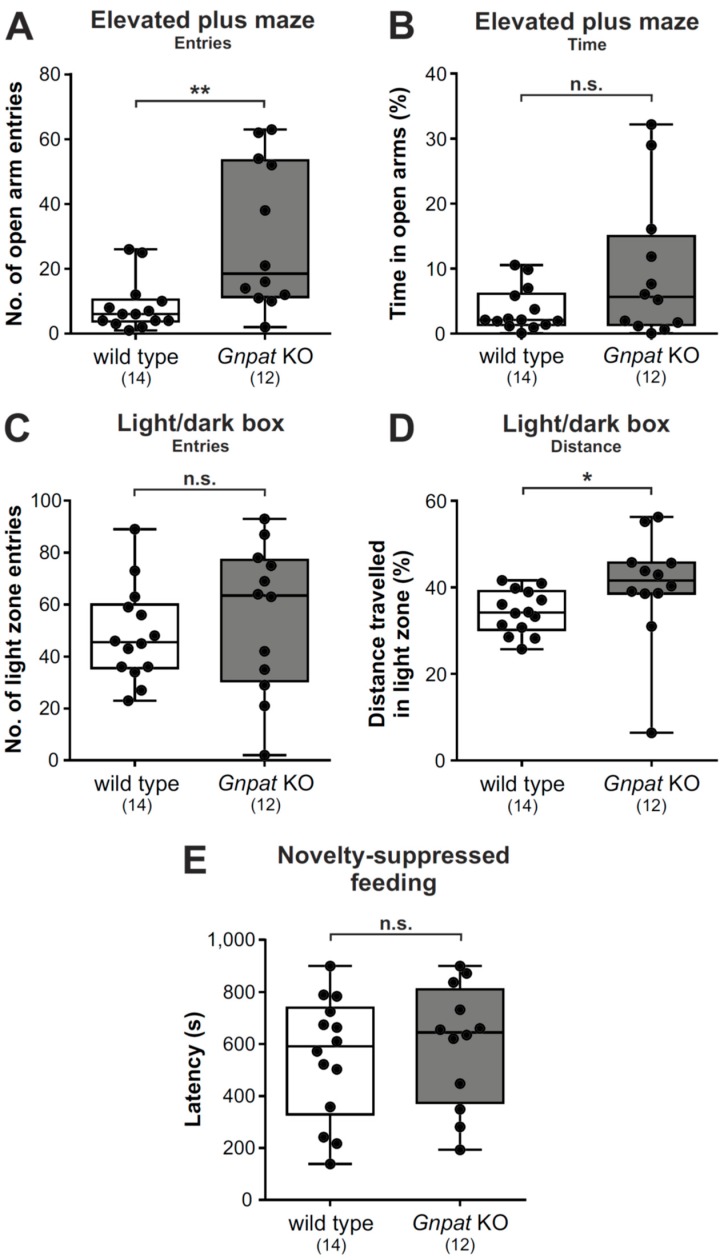
Elevated plus maze and light/dark box testing suggest slightly reduced levels of anxiety in *Gnpat* KO mice: WT and *Gnpat* KO mice were tested in the elevated plus maze paradigm and the number of entries (**A**) as well as the time spent in open arms (**B**) were analyzed. Number of entries (**C**) and distance travelled in the light zone (**D**) by WT and *Gnpat* KO mice are shown as percentages of the total distance travelled during the trial in the light/dark box test. (**E**) Latency in the novelty-suppressed feeding test in WT and *Gnpat* KO mice is depicted. Data are shown as box plots together with individual data points, and whiskers indicate minimal and maximal values (all panels). The number of tested animals is given in brackets. Statistical analysis was done using the Mann–Whitney U test (Figure 4A–D) and two-tailed Student’s *t*-test (Figure 4E). ** *p* < 0.01, * *p* < 0.05; *n.s.*, not significant.

We next used three different paradigms (the elevated plus maze, the light/dark box, and the novelty-suppressed feeding test) to examine anxiety-related behavior. Entries into the open arms in the elevated plus maze serve as an indicator of reduced anxiety [30]. In our test cohort of WT and *Gnpat* KO mice, we revealed significantly increased counts of open arm entries for ether lipid-deficient mice as compared to WT animals (Figure 4A; Figure S3) without alterations in the amount of time spent in the open arms (Figure 4B; Figure S4). This finding may in part be a consequence of the hyperactive phenotype and the motor deficits in *Gnpat* KO mice [21] preventing their free movement within the open arms without falling off, which may prompt them to exit open arms quickly after entering.

In the light/dark box, increased exploration of the light zone has been linked to reduced anxiety [31,32]. Conversely to the findings in the elevated plus maze, *Gnpat* KO mice did not enter the light zone more often than WT littermates (Figure 4C). However, automated tracking revealed that a significantly increased percentage of the exploration distance of *Gnpat* KO animals was covered in the light zone as compared to WT mice (Figure 4D).

Finally, we performed the novelty-suppressed feeding test (Video S3), which assesses hyponeophagia, the anxiety-related reduced feeding behavior in response to a novel environment. Here, we did not record any genotype difference in the latency to consume a food pellet in the test arena (Figure 4E).

### 2.5. No Alteration in the Tail Suspension Test but Considerably Reduced Immobility in the Forced Swim Test in Gnpat KO Mice

**Figure 5:**
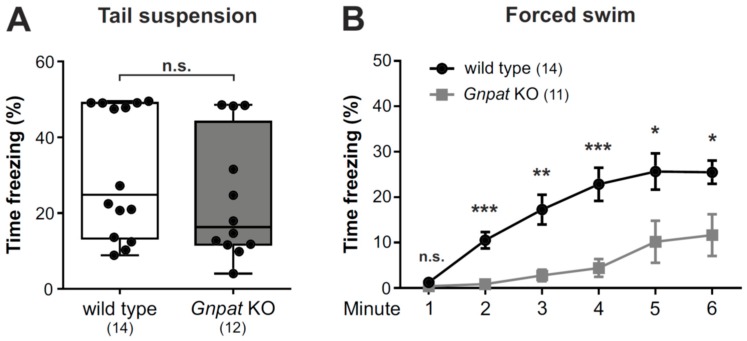
Prominent changes in the forced swim paradigm but not the tail suspension test in ether lipid-deficient animals: (**A**) WT and *Gnpat* KO mice were examined in the tail suspension test, and the duration of the freezing response is shown as box plot with whiskers indicating minimal and maximal values. Statistical analysis was performed using two-tailed Student’s *t*-test. (**B**) The duration of immobility of WT and ether lipid-deficient mice was quantified in each minute of the forced swim test. Results are shown as mean ± standard error of the mean (SEM), and statistical analysis was performed using two-tailed Student’s *t*-tests followed by Holm–Sidak correction for the repeated measurements. The number of tested animals is given in brackets. *** *p* < 0.001, ** *p* < 0.01, * *p* < 0.05; *n.s.*, not significant.

To examine the phenotype of ether lipid-deficient mice in paradigms relevant for depression, we first employed the tail suspension test. Here, the adoption of passive coping reflected by the percentage of time spent immobile is interpreted as depression-like behavior [33]. In our cohort of animals, no differences between the genotypes in the percentage of time spent immobile was observed in this test (Figure 5A).

In contrast, in the related forced swim test, where depression-like behavior is scaled by the evaluation of immobility displayed when mice are put into a water-filled beaker without escape option, substantial differences between WT and ether lipid-deficient animals were detected with *Gnpat* KO mice presenting a statistically highly significant reduction in the percentage of time spent immobile (Figure 5B; Figure S5). However, these results may have to be interpreted against the background of the known overall heightened activity in these mutants.

### 2.6. Ether Lipid-Deficient Mice Have Substantially Reduced Freezing Responses in the Contextual and Cued Fear Conditioning Paradigms

**Figure 6:**
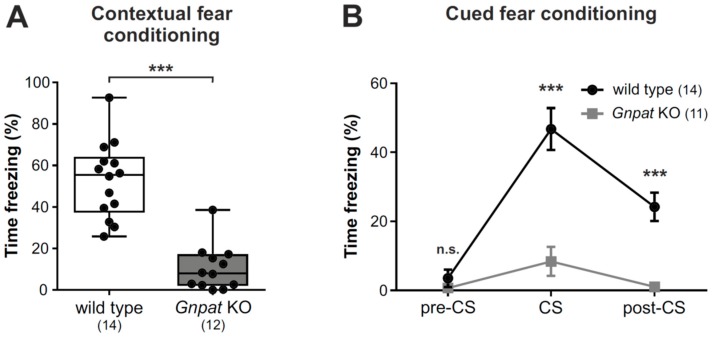
Fear conditioning reveals considerable impairments in contextual and cue learning upon ether lipid deficiency: (**A**) The contextual fear conditioning paradigm was applied to WT and *Gnpat* KO mice, and the duration of the freezing response is shown as box plot, with whiskers indicating minimal and maximal values. Statistical analysis was performed using two-tailed Student’s *t*-test. (**B**) WT and *Gnpat* KO mice were exposed to the cued fear conditioning paradigm, and the duration of the freezing response was quantified before (“pre-CS”), during (“CS”), and after (“post-CS”) the conditioned stimulus (CS). Data are shown as mean ± SEM, and statistical analysis was performed using Mann–Whitney U tests with Bonferroni correction for the repeated measurements. The number of tested animals is given in brackets. The findings displayed in Figure 6A have been published previously with a smaller cohort of WT and *Gnpat* KO mice [11]. *** *p* < 0.001; *n.s.*, not significant.

We next used the contextual and cued fear conditioning procedures to assess associative emotional learning in *Gnpat* KO mice to complement our previous report on contextual fear conditioning in these mice [11]. We now present data from an extended cohort, thus confirming our earlier results demonstrating that *Gnpat* KO mice show markedly reduced freezing responses compared to WT animals in the contextual paradigm (Figure 6A).

Similarly, in the cued fear response test, *Gnpat* KO mice showed a substantially diminished freezing response in the presence of the conditioned stimulus (CS) in comparison to WT mice (Figure 6B; Figure S6; Video S4). Again, these findings have to be carefully evaluated under the consideration of the overall phenotype of ether lipid-deficient mice, which includes a display of hyperactivity.

## 3. Discussion

In the current study, we examined in detail the behavioral consequences of an inborn deficiency in ether lipid biosynthesis in mice, thus complementing our previous studies demonstrating hyperactivity, impaired social behavior, and increased stereotypy (rearing) in this mouse model [11]. Here, we confirm and extend the observations of compromised social interaction and fear conditioning and reveal marked alterations in marble burying and nestlet shredding as well as differential performance in paradigms related to anxiety and depression in ether lipid-deficient animals in comparison to WT controls.

Already in previous papers, we speculated about a potential relation between ether lipid deficiency and neurodevelopmental disorders, particularly ASD [11,13]. This hypothesis is now supported by various observations, including the identification of reduced plasmalogen levels in the blood of autistic patients [34], and reports about clinical diagnoses of ASD and ADHD in patients with RCDP [17,35]. The genetic link between *PEX7* and ASD, as proposed in two recent publications [17,36], further adds to these findings. Even more, *de novo* mutations in another ether lipid-metabolizing enzyme, alkylglycerol monooxygenase, have been identified in autistic patients by two independent studies [37,38]. In the present paper, we built on the hypothesis of an association between ether lipid deficiency and neurodevelopmental disorders and addressed characteristics of these illnesses in *Gnpat* KO mice. Next to testing for social interaction and stereotypic behavior, one of the most frequently used behavioral assays when evaluating the potential presence of autistic features in mouse models is the marble burying paradigm. Even though mostly associated with excessive marble burying, several rodent models of ASD have been reported to show decreased burying behavior [39,40,41,42] as also observed in ether lipid-deficient mice in our study. Remarkably, in one of these studies, decreased marble burying was compared to the restricted interest of autistic patients in their environment [39].

The deficit in marble burying is certainly one of the most striking behavioral alterations detected in *Gnpat* KO mice. We exclude lack of motivation or recognition of the marbles to account for the reduced burying behavior, as *Gnpat* KO animals readily interact with the marbles. Instead, there appears to be a defect in the execution of an inborn behavioral pattern, i.e., digging, which may be a consequence of the impaired neurotransmitter homeostasis and neurotransmission we have described previously [11]. Interestingly, both the pathways associated with anxiety [43] and those associated with repetitive behaviors [44] have been suggested to be involved in burying and digging behavior. Repetitive behavioral patterns have been linked to imbalances in the cortico–basal ganglia–thalamic pathway, which includes the striatum—one of the brain regions in which we have identified a considerable neurotransmitter deficit [11] and which has also been implicated in the regulation of hyperactivity and motor execution [45,46]. However, all our available data point to a more or less ubiquitous neurotransmitter deficit in *Gnpat* KO mice not restricted to certain brain regions, and thus, the consequences for individual neuronal pathways cannot be reliably predicted.

Notably, other genetically altered mouse models that best reflect the behavioral phenotype of *Gnpat* KO mice, as described in our study, mostly involve manipulations of neurotransmitter receptors. For example, mice with a deletion of the gene coding for the β_3_ subunit of the GABA_A_ receptor (gabrb3^-/-^), besides a variety of other deficits, present markedly increased locomotor activity, reduced nestlet shredding, and impaired social interaction [28,47], reminiscent of our observations in *Gnpat* KO mice. Even more similarities can be listed for a mouse model expressing strongly reduced levels of the N-methyl-D-aspartate (NMDA) receptor NR1 subunit (*Grin1^neo/neo^*). Next to hyperactivity, these animals display repetitive behavior—like that also present in *Gnpat* KO mice in the form of continuous rearing (as indicated by the increased vertical movement counts in our previous work [11])—, decreased marble burying, and impaired sociability [42,48,49,50], even though results are conflicting with one study reporting hypersociability rather than impaired social interaction [51]. Remarkably, *Grin1^neo/neo^* mice also show altered anxiety-related behavior, i.e., increased exploration of the open arms in an elevated zero maze and more time spent in the center of the open field, with both features indicating reduced anxiety-like behavior [50,51]. Even though, as discussed above, the interpretation of tests addressing anxiety in the context of hyperactivity is intricate, these findings are in striking accordance with our data gathered in ether lipid-deficient mice. Intriguingly, another mutation in the NR1 subunit leads to a drastically reduced cued and contextual fear conditioning response in mice [52], thus drawing another parallel between animals with NMDA receptor dysfunction and those with a deficiency in ether lipids.

The similarity in behavioral phenotype between *Gnpat* KO mice and the mouse models mentioned above certainly strengthens the hypothesis that a ubiquitous dysregulation of neurotransmission, as reported previously in ether lipid-deficient nervous tissue [11,21,53], may underlie the behavioral alterations revealed in *Gnpat* KO animals. However, as mentioned previously, in view of the complexity of neuronal circuitry throughout the brain and the multitude of brain regions involved in the regulation of behavior, it may be challenging to identify specific regions, neuronal pathways or neurotransmitter systems responsible for the behavioral changes upon ether lipid deficiency.

Noteworthily, a recent publication demonstrated social deficits when shifting the dietary fatty acid composition, specifically the ratio between ω-3- and ω-6-containing fatty acids, in mice [54]. We have shown previously that, in the murine brain, ether lipid deficiency is mainly compensated by increased levels of ω-6-containing phosphatidylethanolamine species, thus shifting the ω-3/ ω-6 balance in the abundant group of ethanolamine phospholipids [55]. Consequently, the ω-3/ ω-6 fatty acid ratio may be a factor contributing to altered social interaction in *Gnpat* KO mice.

Upon assessment of learning and memory, we identified a profound impairment in the response to fear conditioning in *Gnpat* KO mice. Even though we cannot fully exclude an influence of confounding factors, particularly the hyperactivity, in this paradigm, the magnitude of the observed changes suggests a considerable disturbance of associative learning upon ether lipid deficiency. Remarkably, both cued and contextual fear conditioning were strongly affected. Given that, at least in part, different neuronal pathways have been implicated in the two paradigms [56], these findings support the notion of a ubiquitous defect that is not restricted to individual brain regions or neurotransmitter systems in *Gnpat* KO mice.

Interestingly, a role of a deficit in plasmalogens, the most abundant type of ether lipids, has been repeatedly debated in the etiology of Alzheimer’s disease, the most common cause of memory deficit in humans (reviewed in Reference [13]). Compared to the complete ether lipid deficiency in *Gnpat* KO animals, the reported reduction of plasmalogen levels in the brain of Alzheimer’s disease patients is small [57,58]. However, in the context of other pathologic events in the diseased brain, a plasmalogen deficit may unfold more drastic consequences. Our findings in the current study thus add another puzzle piece linking a deficiency in ether lipids with memory impairment, similarly to the one observed in Alzheimer’s disease.

We are certainly aware that the presented behavioral observations may be impacted by other phenotypic hallmarks caused by ether lipid deficiency, including disturbances of the motor system [21], visual impairments [18], or hyperactivity. Also, the proposed general disturbance of neurotransmitter homeostasis and transmission and the resulting compensatory mechanisms in the ether lipid-deficient brain are presumed to impact certain neuronal pathways more heavily than others. This may partly explain task-specific performances of *Gnpat* KO mice (e.g., reduced immobility in the forced swim test but not the tail suspension test) and, at the same time, highlights that some caution is warranted in the interpretation of the results of the individual tests and their interrelationship.

## 4. Materials and Methods

### 4.1. Mice

Mice with a targeted inactivation of the *Gnpat* gene (*Gnpat^tm1Just^,* MGI: 2670462) have been described previously [18]. The strain was maintained on an outbred C57BL/6 x CD1 background, and experimental cohorts with *Gnpat*^-/-^ (KO) and *Gnpat*^+/+^ (WT) littermates were obtained by mating heterozygous animals. Genotypes were determined at weaning by PCR as described previously [18] and confirmed at death. Mice had access to standard chow and water *ad libitum* and were housed in a temperature- and humidity-controlled room with a 12:12 h light–dark cycle and a low level of acoustic background noise at the local animal facility of the Medical University of Vienna.

### 4.2. Behavioral Testing—General Aspects

Adult male mice (littermates, as far as possible) between 3 and 7 months of age were used for all experiments. The restriction to males may pose some limitation to our findings, even though ASD and ADHD, the two human diseases probably best resembling the behavioral phenotype of *Gnpat* KO mice, are more frequently diagnosed in males than in females [59,60]. However, the human disease RCDP is not known to present gender-specific prevalence (as far as it can be evaluated from the limited number of patients). In addition, the hyperactive phenotype is consistently observed also in female ether lipid-deficient mice (own unpublished observation) so that we are highly confident that the main conclusions of our study hold also true for female animals.

Mice were housed individually for at least one week prior to the initiation of experiments and habituated to the testing room for at least one hour prior to each experiment. Tests were conducted in order of increasing stress severity [61] and a minimal inter-test interval of 24 h was observed in all instances. All behavioral experiments were conducted during the light phase of the light–dark cycle. Before starting behavioral testing, all animals were examined for their visual and auditory abilities in order to ensure that potential deficits would not bias subsequent experiments. To this end, reaction to direct exposure to bright light and loud noise (clapping) was tested and animals not reacting to one or both of these stimuli were excluded from further experiments.

### 4.3. Social Interaction and Social Novelty Tests

Social interaction testing was conducted following a previously published protocol in a three-chambered sociability cage (Noldus Information Technologies, Wageningen, The Netherlands) [11,62]. Prior to each trial, the testing arena was cleaned with 70% ethanol and deionized water. For behavioral testing, the test mouse was placed in the central compartment and allowed to freely investigate the arena during a 30-min habituation period. On the following day, the mouse was again placed in the central compartment of the arena, with an unfamiliar age- and sex-matched mouse (“stranger”) in a wired cage (allowing nose contact but no further physical contact) in one side chamber and a “dummy” mouse (inanimate object, black Lego^®^ blocks) in the other. The locations of the unfamiliar mouse and the object were alternated after each test animal. Trial time was 10 min. The unfamiliar “stranger” mouse was habituated to the wire cage before testing and had no previous encounters with the subject mouse.

For social novelty testing, which was performed directly after the social interaction test, the inanimate object was replaced by a second unfamiliar age and sex-matched mouse (“stranger 2”) and a second 10 min trial was conducted.

For recording and analysis of both trials, the Ethovision12XT^®^ program (Noldus Information Technologies, Wageningen, The Netherlands) was used. To quantify the duration spent with either the strangers or the object, a specific area (“sniff zone”) around the wire cages was defined. The time the nose-tip of the subject mouse was inside the sniff zone was used for calculations.

### 4.4. Buried Food Test

Animals were deprived of food for 20 h prior to the test. As described previously [63], a food pellet was buried in a fresh cage approximately 1 cm below the ground and the latency until the mouse held the pellet in its paws to consume it was used for calculations. Maximal trial time was 10 min.

### 4.5. Marble Burying Test

The marble burying assay was performed as described previously [24] with minimal modifications. Briefly, 20 black marbles were arranged in a fresh cage filled with 4–5 cm of bedding material. Mice were placed in one corner of the cage to start the trial, and the observation period was 30 min. Subsequently, the number of displaced marbles and the number of covered marbles (>50% of the marble surface covered by bedding material) was scored by three independent investigators, and the mean was used for calculations.

### 4.6. Nestlet Shredding and Nest Building Tests

Both assays were performed on the basis of previously published protocols [26,64]. Before the onset of both tests, mice were deprived of any nesting material for at least 24 h. For nestlet shredding, mice were supplied with a nestlet (Ancare, Nassau, NY, USA) in their home cage for 30 min. Nestlets were weighed before and after the test, and the weight difference was used for calculations.

For nest building, mice were provided with a nestlet for 48 h and the nests were pictured after 20 and 48 h. Scoring was performed according to a published grading system [64] by two independent investigators blinded to the genotype of the animals, and the average score was used for statistical calculations.

### 4.7. Elevated Plus Maze Test

As described previously [65], mice were placed in a plus-shaped maze elevated 50 cm above the ground, which consisted of two open opposing arms and two closed opposing arms (surrounded by 20 cm high black walls). Upon initiation of the test, all animals were placed in the center area, facing a closed arm. All movements were video-recorded for 5 min and analyzed by software assistance (VideoTrack, ViewPoint, Lyon, France). The number of entries into open arms as well as the time spent in light arms were determined and used as indicator of anxiety-related behavior.

### 4.8. Light/Dark Box Test

Following a standard protocol [31], anxiety-like behavior was determined by placing the mouse in a two-chamber test arena (27.3 × 27.3 cm) consisting of a dark (0 lux) and a light (300 lux) compartment. Initially, all animals were positioned in the dark chamber and allowed to move freely in the entire arena for 6 min. The locomotor activity was measured automatically using a tracking software (Activity Monitor, MedAssociates Inc., St. Albans, VT, USA), and the distance travelled in the light zone as well as the entries into the light zone were calculated.

### 4.9. Novelty-Suppressed Feeding Test

Testing was carried out as described previously [66]. Prior to testing, animals were weighed and food-restricted for 24 h. On the test day, after recording of body weights, a food pellet was placed in the center of a brightly illuminated arena (30 x 50 cm; 800 lux; Video S3) and the latency to the first bite was recorded.

### 4.10. Tail Suspension Test

Experiments were performed according to a previously published protocol [67] using an automated tail suspension system (MedAssociates Inc., St. Albans, VT, USA). Movements were recorded for a total of 6 min by the computational tracking system (Activity Monitor, MedAssociates Inc., St. Albans, VT, USA). The percentage of time spent immobile was calculated and used as indicator for behavioral despair.

### 4.11. Forced Swim Test

Following a published procedure [67,68], animals were placed in a glass beaker (19 × 23 cm) filled with water (22–24 °C) for a total of 6 min. Their movements were video-recorded and analyzed using the VideoTrack software (ViewPoint, Lyon, France). At the end of the experiments, animals were gently dried with a paper towel and kept under a heating lamp until fully dried. The percentage of time spent immobile was determined for each minute of the trial.

### 4.12. Fear Conditioning

Fear conditioning was conducted in designated chambers (MedAssociates Inc., St. Albans, VT, USA) located in sound-proof boxes according to a previously published procedure [69]. Briefly, mice were exposed to an auditory CS (75 dB tone) for 30 s which was temporally paired to an unconditioned stimulus (US; electric foot shock: 0.6 mA, 2 s). Contextual fear conditioning was tested 24 h after the last training day by placing the mice in the same chamber without US or CS presentation. Contextual fear was determined for a period of 5 min by quantifying the percentage of time spent immobile using the near-infrared (NIR) Video Conditioning System for recording (MedAssociates Inc., St. Albans, VT, USA) and Video Freeze software (MedAssociates Inc., St. Albans, VT, USA) for analysis. After a 3-h break, mice were tested for cued fear memory by exposing them to a single CS presentation in a modified context and the percentage of time spent immobile during the CS and the corresponding time period before and after the CS was evaluated.

### 4.13. Study Approval and Ethical Considerations

Animal experiments were carried out in compliance with the 3Rs of animal welfare (replacement, reduction, and refinement), and the number of animals was reduced to the estimated minimum necessary to obtain clear-cut, statistically significant results. The experiments were approved by the Institutional Animal Care and Use Committee of the Medical University of Vienna and the Austrian Federal Ministry of Science and Research (BMWF-5.011/0003-II/10b/2009, BMWF-66.009/0010-II/3b/2014 and BMBWF-66.009/0174-V/3b/2019).

### 4.14. Statistics

Groups of WT and *Gnpat* KO mice were compared by unpaired, two-tailed Student’s *t*-tests or Mann-Whitney U-tests depending on data distribution. Repeated-measures two-way analysis of variance (ANOVA) and two-tailed, paired t-tests were used to analyze social interaction and social novelty tests (Figure 1). Correction for multiple comparisons was performed where applicable. Details on the number of animals and statistical tests used for each experiment can be found in the figure legends.

## 5. Conclusions

In summary, we have performed a detailed behavioral characterization of ether lipid-deficient mice based on previous observations linking ether lipid deficiency to neurodevelopmental disorders like ASD or ADHD. Our results suggest that ether lipid deficiency evokes a neurobehavioral phenotype, which indeed includes features typical of these disorders but, at the same time, is highly complex, manifesting in behavioral alterations also associated with other psychiatric disorders. Even though it may be difficult to pinpoint our results to molecular deficits in individual regions or neuronal circuits, these behavioral data underline that ether lipid deficiency markedly impairs brain function, resulting in a variety of aberrant behavioral patterns. Furthermore, the presented model of complex behavioral changes in ether lipid deficiency may provide a novel tool for selective intervention into specific neurotransmitter systems (e.g., by blocking reuptake of a particular transmitter) in order to gain additional knowledge on the relevance of individual neurotransmitter systems for certain behavioral tasks.

Finally, our data add important facets to the characterization of the phenotype upon ether lipid deficiency. Thus, tests like marble burying or the examination of social behavior can serve as quick and reliable readouts in the assessment of therapeutic strategies against ether lipid deficiency, for which no effective treatment is available so far.

## Supplementary Materials

Supplementary materials can be found at https://www.mdpi.com/1422-0067/20/16/3929/s1.
