# Supplementary material for: Ether Lipid Deficiency in Mice Produces a Complex Behavioral Phenotype Mimicking Aspects of Human Psychiatric Disorders
Source: Int J Mol Sci. 2019 Aug 13;20(16):3929. doi: 10.3390/ijms20163929 (PMC6720005; doi:10.3390/ijms20163929)
Supplement: Supplementary file 1 [file ijms-20-03929-s001.zip › Supplementary Material Legends.docx]

**Supplementary Material**

**Figure S1.** *The buried food test indicates roughly normal olfactory function in ether lipid-deficient mice.* The latency of WT and *Gnpat* KO mice until perceiving a food pellet buried approximately 1 cm below the surface was recorded and data are shown as box plots together with individual data points. Whiskers indicate minimal and maximal values and statistical analysis was performed using Mann-Whitney U-test. The number of tested animals is given in brackets. *n.s.*, not significant

**Figure S2.** *Normal nest building behavior in Gnpat KO mice.* Nest building was evaluated in WT and *Gnpat* KO mice and representative pictures are shown in (A). Scoring was performed after 20 (t20, B) and 48 (t48, C) hours using a scale ranging from 1 (worst) to 5 (best). Data are shown as box plots together with individual data points and whiskers indicate minimal and maximal values. The number of tested animals is given in brackets. Statistical analysis was performed using Mann-Whitney U-test. *n.s.*, not significant

**Figure S3.** *Representative tracks derived from trials of WT and Gnpat KO mice in the elevated plus maze.* Images show the outline of the elevated plus maze with horizontal bars representing the open arms and the vertical bars representing the closed arms. The cross-shaped outline in the middle indicates the center area. Activity of the animals is displayed as either a red line, indicating major or ambulatory movements, or a green line, indicating small movements.

**Figure S4.** *Association between number of entries and time spent in open arms in the elevated plus maze.* WT and *Gnpat* KO mice were tested in the elevated plus maze paradigm (see also Figure 4A, B) and the number of entries and the time spent in open arms were correlated for each individual animal. Correlation analysis (Pearson r) between the two metrics did not yield a statistically significant result (P=0.66).

**Figure S5.** *Raw data derived from the forced swim test.* The duration of the freezing response of WT and ether lipid-deficient mice was quantified in each minute of the forced swim test. The data shown here are the raw data underlying Figure 5B. Lines connect the mean values for each genotype.

**Figure S6.** *Raw data derived from cued fear conditioning.* WT and *Gnpat* KO mice were exposed to the cued fear conditioning paradigm and the duration of the freezing response was quantified before (“pre-CS”), during (“CS”) and after (“post-CS”) the conditioned stimulus. The data shown here are the raw data underlying Figure 6B. Lines connect the mean values for each genotype.

**Video S1.** *Typical behavior of WT mice in the marble burying paradigm.* As exemplified by the short clip, WT animals regularly engage in digging behavior resulting in the majority of marbles displaced and/or buried.

**Video S2.** *Typical behavior of Gnpat KO mice in the marble burying paradigm.* As exemplified by the short clip, *Gnpat* KO mice interact with the marbles by sniffing and touching but hardly displace or bury them.

**Video S3.** *Example of a trial in the novelty-suppressed feeding test.* The presented video exemplifies testing of a *Gnpat* KO mouse in the novelty-suppressed feeding test.

**Video S4.** *Example of a fear conditioning trial.* The fear conditioning paradigm followed the protocol: 60 sec blank (no auditory stimulus), 30 sec CS (tone), 120 sec blank. The two windows of the clip show one WT and one *Gnpat* KO mouse. Note that the depicted WT mouse shows a prominent freezing reaction in response to the CS, whereas the *Gnpat* KO animal hardly reacts to the CS.
