# Supplementary figures and images for: Ether Lipid Deficiency in Mice Produces a Complex Behavioral Phenotype Mimicking Aspects of Human Psychiatric Disorders
Source: Int J Mol Sci. 2019 Aug 13;20(16):3929. doi: 10.3390/ijms20163929 (PMC6720005; doi:10.3390/ijms20163929)

Figure S1

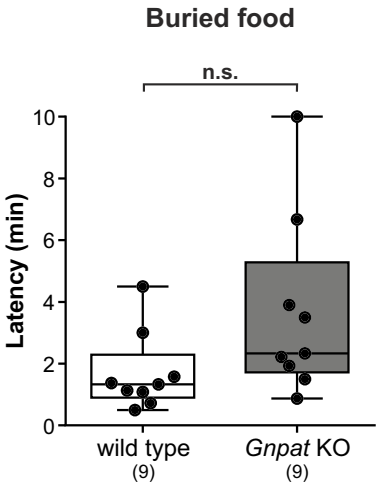

Supplement: Supplementary file 1 [file ijms-20-03929-s001.zip › Figure S1.pdf]

Figure S2

**A**

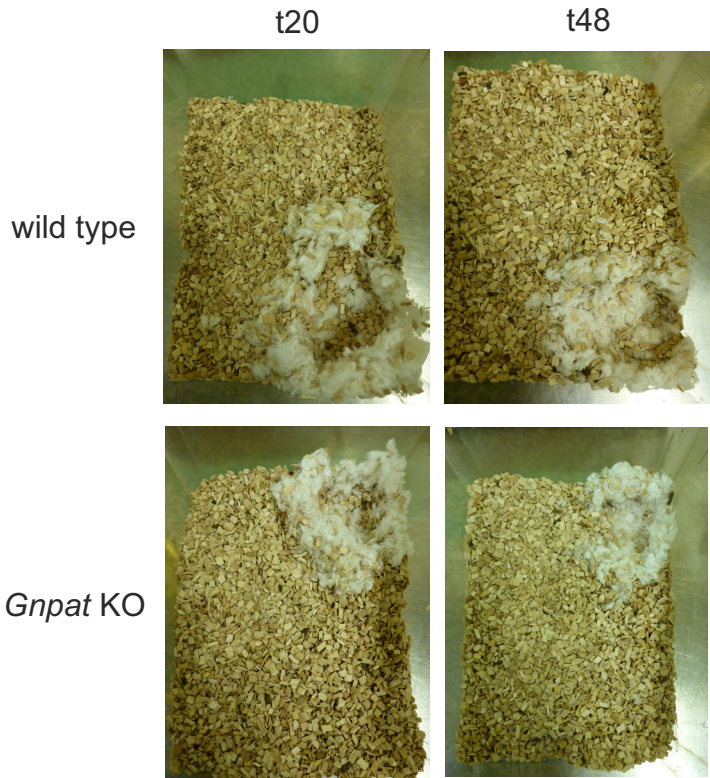

**B**

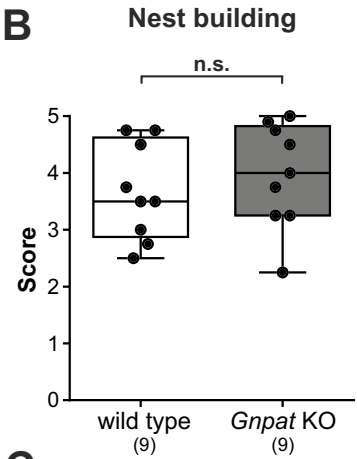

**C**

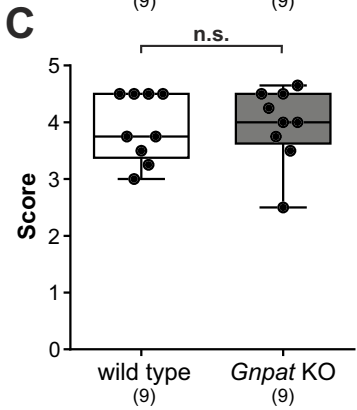

Supplement: Supplementary file 1 [file ijms-20-03929-s001.zip › Figure S2.pdf]

Figure S3

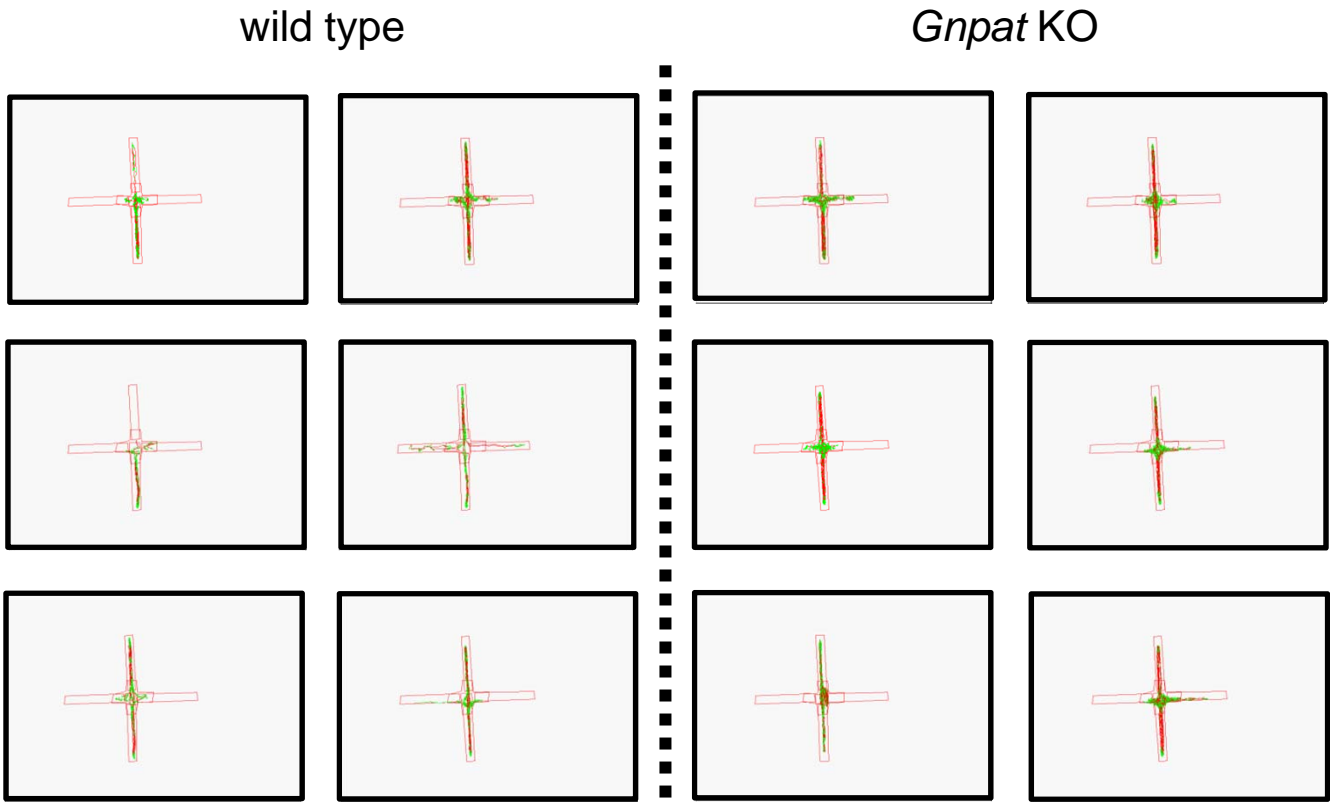

Supplement: Supplementary file 1 [file ijms-20-03929-s001.zip › Figure S3.pdf]

Figure S4

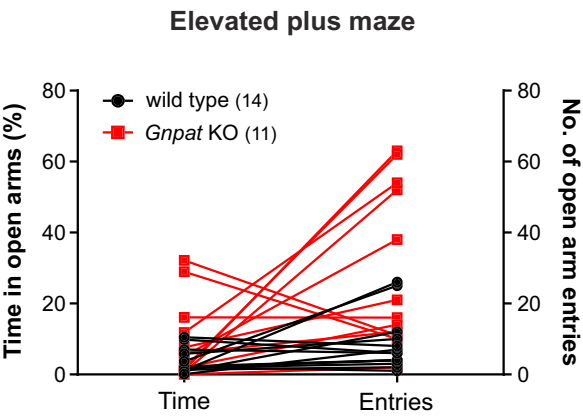

Supplement: Supplementary file 1 [file ijms-20-03929-s001.zip › Figure S4.pdf]

Figure S5

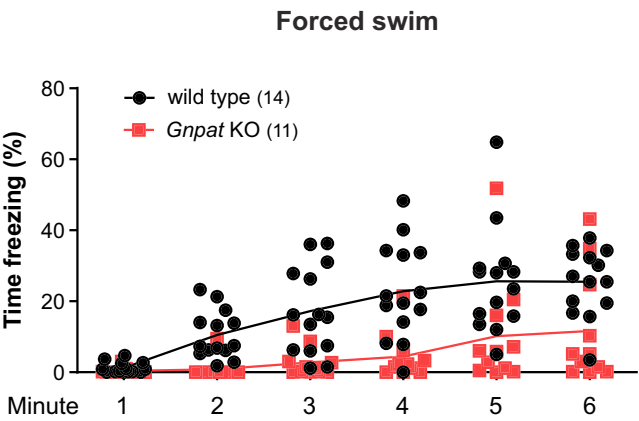

Supplement: Supplementary file 1 [file ijms-20-03929-s001.zip › Figure S5.pdf]

Figure S6

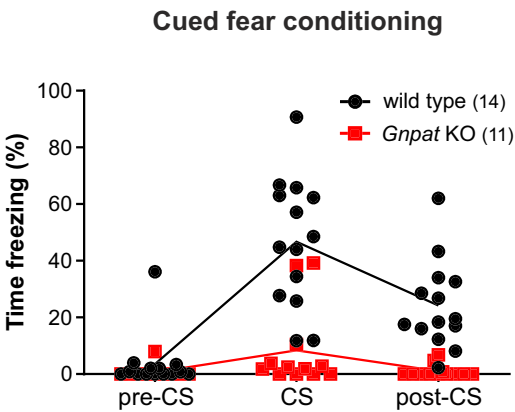

Supplement: Supplementary file 1 [file ijms-20-03929-s001.zip › Figure S6.pdf]
